# Local-time averaged maps of ${H_{3}}^{+}$ emission, temperature and ion winds

**DOI:** 10.1098/rsta.2018.0405

**Published:** 2019-08-05

**Authors:** Tom S. Stallard, Kevin H. Baines, Henrik Melin, Thomas J. Bradley, Luke Moore, James O'Donoghue, Steve Miller, Mohammad N. Chowdhury, Sarah V. Badman, Hayley J. Allison, Elias Roussos

**Affiliations:** 1 Department of Physics and Astronomy, University of Leicester, University Road, Leicester LE1 7RH, UK; 2 Atmospheric Oceanic and Space Science University of Wisconsin-Madison, 1225 W Dayton St, Madison, WI 53706, USA; 3 Center for Space Physics, Boston University, 725 Commonwealth Avenue, Room 506, Boston, MA 02215, USA; 4 Institute of Space and Astronautical Science, JAXA, Yoshinodai 3-1-1, Chuo-ku, Sagamihara, Kanagawa 252-5210, Japan; 5 Department of Physics and Astronomy, University College London, Gower Street, London WC1E 6BT, UK; 6 Department of Physics, Lancaster University, Bailrigg, Lancaster LA1 4YW, UK; 7 GFZ German Research Centre for Geosciences, Potsdam, Germany; 8 Max Planck Institute for Solar System Research, Justus-von-Liebig-Weg 3, D-37077, Goettingen, Germany

**Keywords:** Saturn's aurora, Saturn's ionosphere, ionosphere–magnetosphere coupling, ionosphere–thermosphere coupling, infrared astronomy

## Abstract

We present Keck-NIRSPEC observations of Saturn's ${H_{3}}^{+}$ aurora taken over a period of a month, in support of the Cassini mission's ‘Grand Finale’. These observations produce two-dimensional maps of Saturn's ${H_{3}}^{+}$ temperature and ion winds for the first time. These maps show surprising complexity, with different morphologies seen in each night. The ${H_{3}}^{+}$ ion winds reveal multiple arcs of 0.5–1 km s^−1^ ion flows inside the main auroral emission. Although these arcs of flow occur in different locations each night, they show intricate structures, including mirrored flows on the dawn and dusk of the planet. These flows do not match with the predicted flows from models of either axisymmetric currents driven by the Solar Wind or outer magnetosphere, or the planetary periodic currents associated with Saturn's variable rotation rate. The average of the ion wind flows across all the nights reveals a single narrow and focused approximately 0.3 km s^−1^ flow on the dawn side and broader and more extensive 1–2 km s^−1^ sub-corotation, spilt into multiple arcs, on the dusk side. The temperature maps reveal sharp gradients in ionospheric temperatures, varying between 300 and 600 K across the auroral region. These temperature changes are localized, resulting in hot and cold spots across the auroral region. These appear to be somewhat stable over several nights, but change significantly over longer periods. The position of these temperature extremes is not well organized by the planetary period and there is no evidence for a thermospheric driver of the planetary period current system. Since no past magnetospheric or thermospheric models explain the rich complexity observed here, these measurements represent a fantastic new resource, revealing the complexity of the interaction between Saturn's thermosphere, ionosphere and magnetosphere.

This article is part of a discussion meeting issue ‘Advances in hydrogen molecular ions: H_3_^+^, H_5_^+^ and beyond’.

## 1. Introduction

In August 2017, we planned and executed an ambitious series of observations from Earth, using the 10 m Keck telescope to map out the physical conditions in Saturn's aurora for the first time. On 15 September 2017, after 13 years orbiting Saturn, the Cassini spacecraft finally plunged into the atmosphere of that Giant Planet, ending one of the most successful space missions in history. In its final months of operation, Cassini was shifted into a highly inclined orbit to start its ‘Grand Finale’, a sequence of passes that brought the spacecraft over the polar region of the planet, skimming ever closer to the equatorial ionosphere. This orbit allowed an unprecedented view of the auroral region by the spacecraft's imaging and spectroscopic cameras, as well as providing *in situ* measurements from its magnetic fields and particles instrumentation. As a result, the ‘Grand Finale’ provided a unique opportunity to observe the connection between Saturn's atmosphere, ionosphere and magnetosphere. In order to support this once in a lifetime view of Saturn, we planned and executed an ambitious series of observations from Earth, using the 10 m Keck telescope to map out the physical conditions in Saturn's aurora for the first time.

Before we can review our past understanding of Saturn's aurora, it is important to constrain what we mean by ‘aurora’, especially since discussion during the meeting revealed that the term is neither scientifically well defined, nor well understood by researchers in the broader scientific community. In origin, it was initially used to describe an ‘odd phenomena’ observed in the Earth's night sky [1], named by Pierre Gassendi because it was usually seen in the northern part of the sky, resembling the northern dawn [2]. Many dictionary definitions still constrain the term ‘aurora’ as an Earth-bound phenomena, but within the scientific literature, it has been broadened to include a wide array of emission generally associated with magnetic interactions. A broader and perhaps more satisfying definition is that planetary auroral emissions are ‘an observable signature of the coupling between the planetary magnetosphere and ionosphere' [3]. This definition fails to capture the recent use of the term ‘aurora’ in describing emission from stars [4], from moons like Io and Europa, without an intrinsic magnetic field [5,6] and from the surface of Mercury [7]. It also fails to exclude emissions produced by coupling between the magnetosphere and the equatorial ionosphere which are not described as aurora, such as the recent low-latitude brightening observed at Saturn, described as ‘Ring Rain’ [8]. However, this definition, which we will use here, does allow a discussion of observations of both ultraviolet auroral emission from Saturn caused by direct excitation of atomic and molecular hydrogen, as well as infrared auroral emission radiated by thermalized ${H_{3}}^{+}$ in Saturn's ionosphere. A fuller discussion of Saturn's aurora is given in Stallard *et al*. [9].

Saturn's main auroral emission forms an approximately circumpolar ring [10,11]. This emission is highly structured, including spots and arcs at the smallest scales measured [12–14]. These occur on both the main aurora itself and in a region a few degrees poleward. These poleward emissions are associated with energetic particle injections in the inner magnetosphere on the dawn side [15,16], and with dayside reconnection at noon [10]. This dayside reconnection is also observed as multiple arcs of poleward extended bifurcated emission on the dusk side [17]. While ultraviolet H and H_2_ emission is very similar in structure to the main auroral ${H_{3}}^{+}$ infrared emission [13], ultraviolet observations show very little emission across the polar cap, while significant poleward ${H_{3}}^{+}$ emission has been observed using both spacecraft [18] and ground-based [19] measurements, forming arc-like structures or broader discs of emission.

The main auroral emission is broadly produced by two separate co-located current systems, both of which are ultimately derived from flows within the planet's upper atmosphere ([20]; and references therein). The first is generally axisymmetric, but is modulated with respect to the Sun and is thus fixed in local time. The second rotates with the planet and is fixed to the planetary rotation period. In what follows, we define the term ‘sub-corotation’ to refer to ion flows that are slower in the line-of-sight than the rotation rate of the planet. In the planetary frame of reference, these give rise to retrograde winds, from planetary east to west (dusk to dawn when viewed from Earth). ‘Super-corotation’ then refers to prograde, west-to-east, winds.

The current system fixed in local time occurs within the ionospheric boundary between the magnetic footprints that link to the inner magnetosphere, on which ionospheric ions should move at velocities 60–80% of rigid planetary co-rotation, and field lines connected to co-rotation breakdown in the middle and outer ring current, which should show significant additional sub-corotation [21,22]. Within the upper atmosphere, the neutral atmosphere acts to accelerate ions back to co-rotation, so that they lead the magnetospheric field lines that flow into the ionosphere. This results in field-aligned currents that in turn produce aurora, driven by the exchange of angular momentum from the planetary atmosphere to magnetospheric plasma. These currents map to the outer magnetosphere and so, while they are broadly axisymmetric and producing an auroral oval at all local times, they are also affected by the Solar Wind. This can result in stronger currents on the dawn side of the ionosphere, associated with activity within the magnetospheric tail region [11].

The statistical mean brightness of the main auroral emission is brighter on the dawn side [23], driven by periods of ‘intermediate’ enhancement in the Solar Wind field strength and density, which results in significant ‘dawn brightening’ [24]. Prolonged periods of dusk side brightening have also been observed [25], suggesting there is significant complexity in the generation of these sub-corotationally driven aurora.

Ground-based observations have not revealed the ionospheric flows predicted by modelling. Early observations revealed a polar ionosphere that significantly sub-corotates by approximately 30% of the planet's rotation rate [19], which broadly agreed with initial modelling [21]. However, the initial breakdown in ionospheric co-rotation occurs significantly equatorward of the main auroral emission, mapping to the breakdown in co-rotation observed in the magnetosphere, driven by mass-loading by volcanic water from Enceladus [26,27]. This initial breakdown is co-located with a significant ionospheric brightening, most likely the in-fall of water ions from the Enceladus torus, resulting in a similar enhancement as that seen in Saturn's equatorial ionosphere, driven by Ring Rain from Saturn's rings [8]. However, poleward of this breakdown, the ionospheric sub-corotation sees no sharp boundaries across the auroral region, instead showing a gradual increase in sub-corotation into the polar region [28,29]. Recent analysis of observations using adaptive optics also showed no clear velocity gradients near the main auroral oval [30].

Poleward of the main auroral emission, the magnetic field lines within the ionosphere reach a boundary, beyond which the field lines are connected to the magnetic field trapped within the Solar Wind. These field lines are considered ‘open’ to the Solar Wind, opening through reconnection near noon, moving slowly anti-sunward across the pole then closing on the midnight side. Unlike Earth, where this process takes a few hours, it takes the Solar Wind several Saturnian days to cross the pole, so that a partial co-rotation is imposed on these open field lines [31]. This twists up the magnetospheric tail lobes into approximately cylindrical shells of open field lines, with the most recently opened field lines on the outer edge of the tail, and the longest open field lines at the cylinder's core. Ion wind measurements in Saturn's polar region appear to provide evidence of this older core of open field lines. While the majority of the polar region appears to sub-rotate significantly, a central region is often observed to be fully co-rotational, resulting in a ‘three-tier’ velocity structure [29].

Observations of the ionospheric winds during a major compression in the Solar Wind seem to support this theory. Following the impact of the compression on Saturn's magnetosphere, the entire polar region was observed to sub-corotate. This loss of a central co-rotation region is explained by the sudden reconnection of the entire magnetotail, resulting in the removal of these old, twisted field lines [32]. This large-scale closing of field lines was originally hypothesized to explain the dramatic infilling of the entire dawn side of the polar cap observed during major Solar Wind compressions [33]. However, recent observations of ion winds within the polar region using adaptive optics have shown that this central region may be dominated by noon–midnight flows, significantly complicating this argument [30].

All these local-time orientated auroral emissions are overlain by a separate current system that has been revealed in the periodic magnetic oscillations seen throughout Saturn's magnetosphere. These planetary period oscillations have been directly associated with Saturn's variable rotation rate, as calculated by radio emissions [34]. Observations show that they are driven by two independent rotating systems of field-aligned currents in the northern and southern hemispheres, consisting of a cross-polar current that is linked with the surrounding magnetosphere via field-aligned currents, with a layer of downward currents on one side of the pole and a layer of auroral generating upward currents on the other. These currents overlap those generated within the outer magnetosphere, enhancing and reducing the auroral emission, depending upon the planetary period phase [20,35]. Both magnetospheric observations and modelling implies that this rotating current system is driven by processes occurring within the atmosphere [20,36–38], with a neutral twin-cell vortex positioned over the auroral region, driven by thermal heating, rotating at a rate that then becomes the measured rotation rate within the magnetosphere. This neutral twin-cell vortex drives currents outwards from the ionosphere into the surrounding magnetosphere, an inversion of every other known auroral current system within the solar system. Such a neutral twin-cell vortex is predicted to be associated with very significant thermospheric temperatures, which may be localized in the planetary phase [39].

The majority of ${H_{3}}^{+}$ in the ionosphere lays significantly below the region where local-thermal equilibrium breaks down, meaning that measurements of emission from ${H_{3}}^{+}$ are largely thermalized [40]. But there are observational constraints. Saturn has a relatively poor level of methane absorption in its lower atmosphere compared with both Jupiter and Uranus, so that there are more wavelength regions where there is a significant amount of reflected sunlight. This means that higher resolution instrumentation is needed to resolve ${H_{3}}^{+}$ line emission from the underlying planetary signal. As a result, spacecraft measurements of the thermospheric temperatures are more limited—the Cassini VIMS instrument can only make accurate temperature measurements when observing over very small scales on the planet [13] or above the planet's limb [41]. From Earth, higher spectral resolutions allow ${H_{3}}^{+}$ lines to be separated from the background reflected sunlight, but poorer spatial resolution and the relatively weak ${H_{3}}^{+}$ brightness means it is difficult to observe any spatial variability. Past temperature measurements have shown that Saturn's average auroral temperature can vary significantly, ranging widely between 360 K and 590 K [30,32,42–45].

Observations of the northern auroral region have shown that there can be a temperature difference of greater than 100 K between the noon and midnight sides of the main auroral oval and that, as the planet rotates, the observed temperatures rotating through the planet's meridian can also vary by up to 150 K over only 30 min. This is approximately 20 degrees of rotation, suggesting relatively small-scale temperature structure within the auroral region [45]. High spectral resolution observations of Saturn's auroral region have revealed small-scale temperature increases of approximately 30 K, which have been loosely associated with a significant polar arc inside the main oval, perhaps suggesting that these bright arcs, only seen in the infrared, might be a manifestation of heating within Saturn's thermosphere [30].

In summary, our understanding of the ionosphere and thermosphere of Saturn lags behind the detailed measurements made of Saturn's magnetosphere by Cassini. Our understanding is limited by our ability to spatially resolve emission from the auroral region, making it difficult to measure the ion currents that flow through the ionosphere or the structure of the underlying thermospheric temperatures that are either driven by these aurora, or potentially drive them. In order to better measure these values, it is essential to increase our measured signal to improve the observed signal-to-noise on the planet, as well as bettering our positioning and observing the aurora in two dimensions, so we can map out the ${H_{3}}^{+}$ accurately across the entire auroral region, a strategy that has greatly improved our understanding of Jupiter's ionosphere [46,47].

## 2. Data

The final orbits of Cassini's Grand Finale occurred approximately every 6.5 days until the mission ended with Cassini crashing into Saturn's equatorial region on 15 September 2017, as listed in table 1. During this time, the spacecraft took a series of remote observations of Saturn's auroral region. Investigation of these observations are in the early stages, but they have provided unprecedented high-resolution measurements of the ${H_{3}}^{+}$ aurora and a direct measure of the ultraviolet aurora during the final stages of the mission (e.g. [48]). Unfortunately, these observations are typically made in the days before or after the time of closest approach and are often at too high a resolution to provide a broad-scale comparison to our data. However, there was a significant Hubble Space Telescope campaign made during the Grand Finale period that does provide images of Saturn's combined H and H_2_ aurora during February and September 2017 [49]. Three sets of observations fell in the same period as our Keck observations. On 25 July between 15.51 and 17.26, the UV aurora was relatively weak, with a narrow dawn-enhanced emission and a spot of enhanced emission near midnight. On 7 August between 13.44 and 15.19, the aurora was again fairly weak, brightening a little between the observations, with enhancement at both dawn and dusk. On 14 August, a single image at 06.14 showed a very strongly enhanced aurora, brighter at dawn, in a spiral shape on the dusk side and a very bright cusp-like emission in the dawn–noon sector.

**Table 1.** Times of ring crossing, apoapsis or impact into Saturn for each of the final orbits of Cassini, given in UT propagated over the light travel time to Earth.

orbit	285	286	287	288	289	290	291	292	293 (final)
date	July 25	Aug 1	Aug 7	Aug 14	Aug 20	Aug 27	Sep 2	Sep 9	Sep 15
UT (Earth)	20.12	07.23	18.37	05.41	16.43	03.41	14.39	01.41	11.54

No direct Solar Wind measurements of these conditions outside the magnetopause were made by Cassini during this period, and so we have used observations from the MIMI/LEMMS energetic charged particle detector on the Cassini spacecraft to provide some context. As discussed in Roussos *et al*. [50] (and references therein), the LEMMS instrument can detect solar energetic particles that penetrate Saturn's magnetosphere in its P2–P9 channels. These are mostly MeV protons, and transients of the solar energetic particles are linked with the arrival of interplanetary coronal mass ejections (ICMEs) or corotating interaction regions (CIRs) (e.g. [51]). Additionally, background noise in the LEMMS electron channels can be used, in the right conditions and with the right filtering, to provide a measure of galactic cosmic rays. These galactic cosmic rays are predominantly protons with energy ranges of several hundred MeV to 1 GeV. These are from sources external to the heliosphere and are modulated by variations in the interplanetary magnetic field, such that solar periodicities related to CIRs are clearly identifiable. Additionally, ICMEs will cause a sharp decrease in their detection followed by a slow exponential recovery over several days in a process known as a Forbush decrease [52].

**Figure 1.**
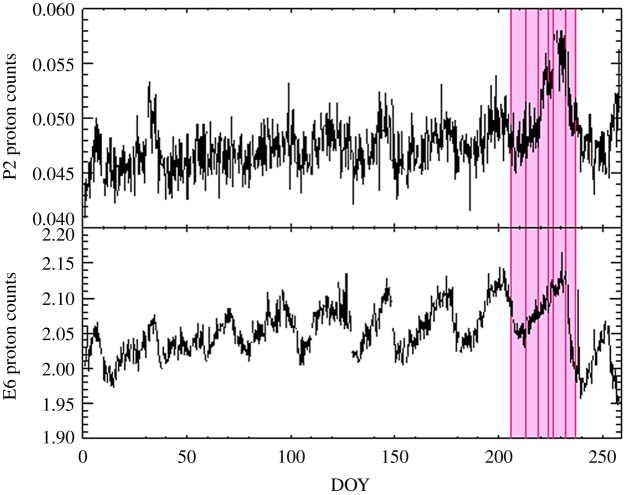
Cassini LEMMS measurements from the P2 and E6 channels. The P2 (2.3–4.5 MeV protons) channel shows the detection of solar energetic proton counts per second and increases in detected protons can be used as an indicator of the arrival of coronal mass ejections. The E6 (1.6–21 MeV electrons) channel shows greater than 120 MeV galactic cosmic ray proton counts per second (once electrons have been filtered)—here, stronger Solar Wind conditions prevent these galactic protons penetrating, and so Solar Wind conditions associated with co-rotating interaction regions are identified by marked decreases in count rate detection. The dates of our observations are also noted (vertical pink lines and shaded region). (Online version in colour.)

Data from LEMMS are 6 h averaged in this study to increase the signal-to-noise ratio similarly to Roussos *et al*. [50,53], though here we have removed spurious enhancements in the averages due to instrument effects. Six-hour averaged count rates from the P2 channel (2.3–4.5 MeV protons) showing solar energetic particle detections are plotted in figure 1 over the course of 2017, cut off at the end of the Cassini mission. An increase in the counts by a factor of approximately 1.5 during the interval of interest for this study, starting on 3 August (day 215), peaking between 16 and 20 August (days 228–232) and ending on 28 August (day 240), has previously been identified as signatures of an ICME by Roussos *et al*. [53]. Figure 1 then shows 6 h averaged count rates from the E6 channel (1.6–21 MeV electrons). This channel has a multi-species response, and while designed to detect greater than 1.6 MeV electrons, it can also respond to greater than 120 MeV protons—by filtering the data, these high energy protons can be isolated, providing a measure of galactic cosmic rays proton counts [54]. A clear approximately 26-day solar periodicity is clearly in this dataset indicative of CIRs reaching Saturn (see [53] for further discussion). Pertinent to the observations in this study is the Forbush decrease on 20 August (day 232), indicating with the identified solar energetic particle enhancements that Saturn's magnetosphere may have interacted with an interplanetary shock between 16 and 20 August (day 228 and 232).

Our observations on the Keck telescope on Mauna Kea in Hawaii were planned to observe Saturn's aurora at the same time as Cassini flew past the planet within its highly inclined orbit, sweeping through magnetic field lines mapping to the auroral region. Although telescope time allocation had skewed the specific timing of the observations, we were able to observe the planet over a period of more than a month with a cadence of every 2–5 days, as described in table 2. Beyond the scope of the results produced by these measurements, the observations themselves were an unmitigated success. It is rare that observations are unaffected by poor atmospheric conditions and bad weather, yet here all seven nights of data were unaffected by significant high cirrus clouds or atmospheric turbulence, with atmospheric seeing better than 0.7′′.

**Table 2.** Details of the observations used in this study. Dates and times are given in Earth UT. Seeing was measured using stellar spectra. Details of the slit width and step size are given, as these were changed over the observations (but were constant in each individual night). The phase of the northern and southern planetary period magnetic oscillation is provided for context to the current system and thermospheric flows that drive this oscillation [55].

date	25 July	1 Aug	7 Aug	12 Aug	14 Aug	20 Aug	25 Aug
letter	(a)	(b)	(c)	(d)	(e)	(f)	(g)
DOY	206	213	219	224	226	232	237
start time	07.06	06.00	06.35	05.31	05.39	06.24	06.19
end time	09.42	09.19	08.19	07.56	08.02	07.53	07.43
light time	77 m	78 m	78 m	79 m	79 m	80 m	81 m
no. of scans	14	18	10	13	11	8	7
seeing	0.69	0.59	0.69	0.56	0.50	0.60	0.56
slit width	0.432	0.432	0.432	0.432	0.288	0.288	0.288
step size	0.4	0.4	0.2	0.2	0.2	0.2	0.2
mean N phase	146	325	81	99	278	34	70
mean S phase	169	51	220	286	104	289	11

Observations from Earth during the late summer were difficult, as Saturn was moving away from opposition on 16 June. Each night after this date, Saturn set earlier in the evening, so that for the final month of Cassini orbits, we could only observe the planet in the first few hours of the night. This meant we could only observe into the evening, with ending times as late as 2242 Hawaiian Standard Time (HST) at the start of the run, shortening to as early as 20.43 HST on the last scanned night of the run—on the 27 September, our observations ended even earlier in the night, leaving us too little observing time to produce a scan of the auroral region. Our observations were greatly improved by the active participation of the Keck support scientist who worked with us to open the telescope significantly earlier than is typical. This allowed us an hour or two of twilight observations that proved critical in the later stages of the observing run. The final three orbits on 27 August and 2 and 9 September were so late, we were unable to scan the aurora. On 27 August, we aligned the NIRSPEC slit north–south on the planet, and for all three we were able to observe Saturn using the IRTF-iSHELL instrument in a fixed east–west cut, but these observations are not included in this study.

**Figure 2.**
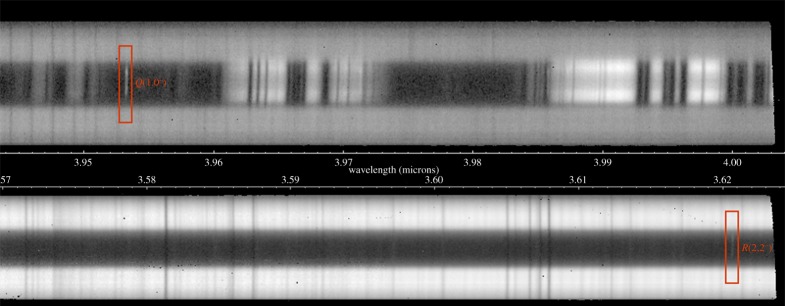
Two infrared spectra of Saturn taken simultaneously by Keck/NIRSPEC. The slit is aligned east–west across the auroral region, so that wavelength is measured in the *x*-direction and east–west spatial information measured in the *y*-direction. At the top is a NIRSPEC order 19 spectrum between 3.94 and 4.01 µm; the two horizontal bars are the continuum reflected sunlight from the rings, between these, in the middle and the right of the spectrum are regions of reflected sunlight from Saturn's lower atmosphere, with surrounding dark regions caused by methane absorption of the sunlight. On the left, the narrow auroral *Q*(1,0^−^) emission line can be seen (highlighted with a red box), with the *Q*(2,0^−^) and *Q*(3,0^−^) lines obscured by reflected sunlight in the middle and right side of the spectrum. At the bottom is a NIRSPEC order 21 spectrum, between 3.58 and 3.63 µm; here the reflected sunlight from the rings is much brighter and the absorption of methane in the lower atmosphere is much more complete. The relatively weak *R*(2,2^−^) line is shown on the right (again highlighted with a red box). To the left of this, the weaker *R*(2,1^−^) line is not seen—at 1/3 the brightness, it is unusually too weak to be seen. (Online version in colour.)

We used the same NIRSPEC settings as in our past Keck auroral observations at Jupiter and Saturn, originally defined in Lystrup *et al*. [56], with the KL filter, an echelle position of 62.02 and a cross-disperser position of 33.69. These were adjusted manually each night, using arc lines, to correct for instrumental variations in the exact echelle and cross-disperser positioning. This resulted in our observations measuring five spectral orders simultaneously (NIRSPEC orders 19–23). In this study, we use two orders (19 and 21), as these give access to the wavelength ranges approximately 3.94–4.01 µm and approximately 3.58–3.63 µm, as shown in figure 2. These spectral regions contain a continuum of reflected sunlight from the rings on either side of the planet (with spatial information distributed on the *y*-axis), some regions where sunlight is reflected off the planet's lower atmosphere and other regions where methane within the atmosphere absorbs this sunlight, leaving the disc of the planet dark. Finally, two relatively bright lines of ${H_{3}}^{+}$ emission are seen; these are the same lines used in previous Saturn auroral studies performed by Keck (e.g. [8,44,45]), the fundamental ${H_{3}}^{+}$ *ν*_2_ *Q*(1,0^−^) line at 3.9530 µm and *ν*_2_ *R*(2,2^−^) line at 3.6205 µm.

**Figure 3.**
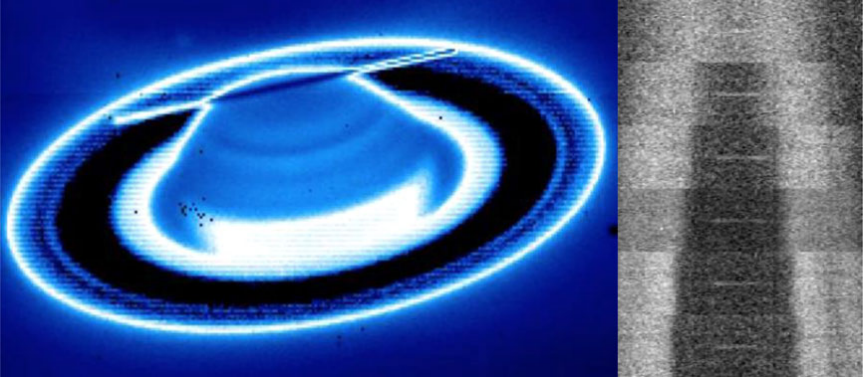
The slit position and scans on Saturn. On the left is a K-band image of Saturn taken using the slit imager on NIRSPEC, showing the alignment of the slit east–west over the northern rotational pole. This slit position is moved perpendicular to the slit across six slit scan positions, shown on the right, covering the auroral region of Saturn. This shows the ${H_{3}}^{+}$ Q(1,0^−^) emission line and the continuum reflected sunlight off Saturn's rings at each of these positions. (Online version in colour.)

On each night, the NIRSPEC slit was scanned across the northern auroral region, as shown in figure 3. NIRSPEC was used with a long-slit configuration allowing us to measure 24′′ of spatial information. The slit width was initially set to 0.432′′ to allow more light into the instrument, but on later nights, we switched to the 0.288′′ slit, as this significantly reduced the Earth's background thermal emission, resulting in an overall increase in signal-to-noise on the planet; these slit widths are described in table 2.

The slit was initially positioned on the planet's northern polar limb, and then a macro was used to control the telescope, first moving away from the planet to take a background ‘sky’ frame, then moving back and taking a sequence of six spectra from Saturn, with the slit moved in equal steps towards the equator through the auroral region. The length of this step (either 0.4′′ or 0.2′′) was constant over an individual night, but was changed for different nights, as described in table 2. The telescope positioning was held by off-axis guiding on a background star and the use of an offset tracking rate that followed Saturn's non-sidereal motion across the sky.

The individual spectra were flat-fielded, straightened in the wavelength and spatial directions using a combination of stellar spectra and emission lines from the Earth's atmosphere, respectively, and flux calibrated using emission from A0 V stars, in the standard way (e.g. [46,57,58]). The spectra were then sky subtracted to remove the Earth's atmosphere. For the majority of the spectra, this was achieved by taking the sky spectra immediately before and after the scan, calculating the relative time (*t*_1_, *t*_2_) between these two sky frames (sky_1_, sky_2_) and the timing of the Saturn spectrum in question (*t*_d_), producing a ratioed sky frame that takes a relative component of light from each sky frame dependent upon the relative time that separates them, using the following equation:

$$\text{sk}y_{\mathrm{Final}}=\left[\mathrm{sk}y_{1}\times \left(\frac{t_{2}-t_{d}}{t_{2}-t_{1}}\right)\right]+\left[\mathrm{sk}y_{2}\times \left(\frac{t_{d}-t_{1}}{t_{2}-t_{1}}\right)\right],$$

where only one nearby sky frame was available, we scaled the sky linearly, where necessary, to account for changes in sky brightness.

Although the initial scan pattern was designed to cover the aurora in equal steps, examination of the spectra showed that the telescope tracking was not always exact and so the predicted location for each spectrum could not be assumed. Instead, we used the reflected sunlight from the planet in each spectrum to model the slit position. First, we isolated the reflected sunlight from the planet by taking the average brightness profile around 3.99 µm, where both planet and rings reflect sunlight, and then subtracted the brightness profile around 3.98 µm, where methane absorption removes all planetary emission, leaving only reflected sunlight from the rings. Secondly, with a pure planetary brightness profile, we could use a brightness threshold of 50% of the peak brightness to identify the limbs of the planet and find the central meridian in each spectrum. Finally, we could perform a least squared fits of the planetary brightness profile against a simple model of reflected sunlight brightness across the planet. The model of reflected sunlight was calculated using a simple decrease in brightness from the equator to the pole as a cosine of double the modulus of latitude. This curve was then normalized between 1.0 at the equator and 0.1 at the pole. Although much simpler than the true equator to pole brightness variation, we found through empirical testing that this produced a reliable fit for the latitudinal location of the slit. In combining the east–west and north–south positioning, it was possible to much more accurately locate the position of each individual slit, as well as calculating the latitude and longitude of each pixel on the planet.

In order to account for this varying location of the slit, we produced a three-dimensional spectral map for each night, consisting of two spatial directions (east–west and north–south) and the wavelength—emission from each spectrum was added to the entire north–south range of positions covered by the slit to ensure that data from different spectra overlapped accurately. As a result, running this position fitting for each individual spectrum not only improved the positioning accuracy of our resultant image, it also allowed a much higher resolution in the north–south direction, as emission was added in a dithered pattern at a sub-slit-length resolution.

Once an individual night was repositioned and co-added, each north–south position was isolated as an individual spectral image, the ${H_{3}}^{+}$ emission line was located and for each east–west position, the Gaussian shape of the emission line was fitted using a six order Gaussian fit (height, position, width and a quadratic background fit) in exactly the same way as in our past measurements of Jupiter and Saturn. This produces a set of two-dimensional images of emission height, position and half-width. In addition to this, we also measured the average reflected sunlight from the rings (around 3.98 µm) and from the planet (3.99 µm emission with ring light removed), producing two images of the rings and planet, respectively.

The total brightness of each emission line of ${H_{3}}^{+}$ is calculated for each point in the images by factoring the fitted Gaussian peak brightness with the Gaussian half-width, using the same technique as in past observations (e.g. [46]).

Using the fitted Gaussian positions for the ${H_{3}}^{+}$ *Q*(1,0^−^) line, it is possible to measure the relative Doppler-shift position at each point on the images. This provides a relative shift in the Gaussian pixel position that can be converted into a relative velocity, as described in past measurements at Jupiter [57,46] and Saturn [19]. In order to provide a zero position for these velocities, on each night we took the mean measured velocity poleward of 75° N and set this to zero. This assumption provides a better zero velocity than the velocity in the line of sight along the central meridian, as has been used in the past, as it better accounts for noon–midnight polar flows, as were recently observed by Chowdhury *et al*. [30]. As with past observations, we have not accounted for Saturn's sub-Earth latitude reducing the observed line-of-sight velocities, instead presenting raw line-of-sight values.

By measuring the ${H_{3}}^{+}$ emission brightness from the *Q*(1,0^−^) and *R*(2,2^−^) lines, it is possible to calculate the ${H_{3}}^{+}$ temperature, column density and total emission in the same way as has been used previously at both Jupiter and Saturn (e.g. [44,47,58]). *Ab initio* calculations of the theoretical emission per molecule of ${H_{3}}^{+}$ provide a theoretical brightness for each line at a given temperature [59]. As a result, a measurement of the relative brightness of two lines resolves out to a specific theoretical ro-vibrational temperature, with changing temperature resulting in a different ratio of brightness between these two lines. This allows us to use the emission from two lines as a measure of ${H_{3}}^{+}$ temperature, assuming local thermal equilibrium.

Since we have measured the line brightness from an entire column of ${H_{3}}^{+}$, we can then divide our observed emission brightness by the molecular ${H_{3}}^{+}$ emission brightness to produce a ${H_{3}}^{+}$ column density. The measured column density is in the line-of-sight, with the depth of column dependent upon the viewing geometry of the planet. We must then account for the line-of-sight enhancement caused by observing ${H_{3}}^{+}$ through the atmospheric depth, which leads to significant limb-brightening. In the past, this calculation has been done using either a depth of atmosphere above the 1 bar level [58] using a cosign function, or by modelling a shell of emission at a specific altitude above the planet [60]. Here, we assume the ${H_{3}}^{+}$ is emitted in a shell of atmosphere between 813 and 1496 km, the half-width full maximum location of the measured ${H_{3}}^{+}$ emission peak [61]. Past observations have assumed a perfect geometry for this line-of-sight enhancement, but the inaccuracy in positioning, caused by poor alignment of data and by seeing in the Earth's atmosphere with significantly smooth the line-of-sight enhancements measured. In order to account for this, we model the line-of-sight enhancement from this shell as a ‘perfect’ two-dimensional image of Saturn, then smooth the enhancement to emulate the smoothing within our dataset. Firstly, we convolve this image by the seeing for each night. Secondly, we use the measured slit position and width to average the line-of-sight enhancement across the entire slit for each spectrum, further averaging these as we build our spectral map from the individual spectra. This results in a smoothed line-of-sight correction that emulates the line-of-sight enhancement measured within the data. We then divide our measured emission by this to produce a calculated vertical column density, as seen from directly above the planet. Although our calculation of the line-of-sight is more complex, the process of correction is the same as we have used in many past observations (e.g. [44,58]).

Since the temperature provides a theoretical measure of the brightness per line per molecule, we can then factor the temperature and column density together to produce a total ${H_{3}}^{+}$ brightness across all thermalized ${H_{3}}^{+}$ lines, known as the ${H_{3}}^{+}$ total emission [58,62]. This is effectively the ${H_{3}}^{+}$ cooling rate from Saturn.

## 3. Results

### (a) Intensity structure

**Figure 4.**
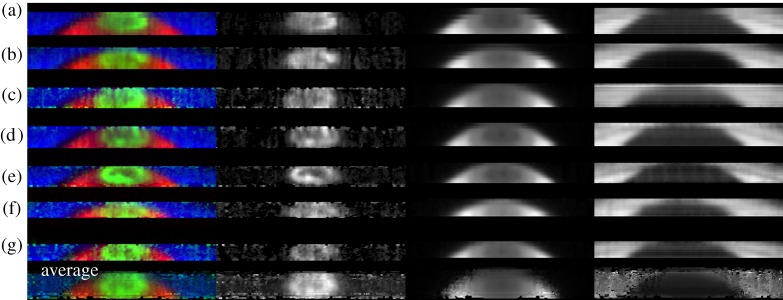
The seven images of Saturn's auroral region. The left column shows the three wavelength regions as a three column image. The second column shows the fitted ${H_{3}}^{+}$ emission (also shown as green in the first column). The third column shows the bright reflected sunlight around 3.99 µm once sunlight reflected off the rings is removed, leaving a pure image of the planet (also shown as red in the left column). The right column shows the reflected sunlight from the rings in a region where methane absorption is strong around 3.98 µm (also shown as blue in the left column). (Online version in colour.)

Figure 4 reveals the detailed morphology obtained by scanning the slit across the auroral region. Because each spectrum can be accurately located on the planet, the resolution is much finer than has been possible in past observations, countering limitations with positioning, resulting in images with a spatial resolution of the seeing on that night, shown in table 2. The measured brightness from three different wavelengths are shown in this figure: the column on the left shows a three-colour image of each of the different wavelengths, shown in the second, third and fourth columns; these are the ${H_{3}}^{+}$ emission from the *Q*(1,0^−^) line, the reflected sunlight from the planet and the reflected sunlight from the rings, respectively. The brightness of each image is scaled linearly between zero and the maximum measured brightness on each night. The individual images are shown in each row, identified by the letters a–g, taken on (a) 25 July, (b) 1 August, (c) 7 August, (d) 12 August, (e) 14 August, (f) 20 August and (g) 25 August, as described in table 2.

The images of reflected sunlight from the planet and rings (columns 3 and 4) provide a direct measure of the accuracy of our fitted positioning, as well as the conditions on the individual nights. Reflected sunlight from the planet clearly shows well-defined bands of atmospheric clouds including the northern polar hood and the much smaller and darker northern polar vortex, a region approximately 1 degree wide at the planet's tropospheric pole, which is especially clear in images (d) and (e). The images of the rings also show similarly high spatial resolutions, with the Cassini division again clearest in images (d) and (e). This suggests we can be confident that we are able to observe the signature of the ${H_{3}}^{+}$ aurora across Saturn's pole at an unprecedented level of spectral and spatial resolution (compared with, for example, [63]).

The brightest ${H_{3}}^{+}$ emission is located around the poles between colatitudes 10° and 15°, latitudes 75° to 80° N (figure 4). Several nights have a clearly identifiable auroral oval on both the dawn and dusk sides (b, d, e, g), but the ${H_{3}}^{+}$ emission is noisy and complex on 20 August (f), either as a result of variable aurora or poor visibility. Several nights also appear to have a thin polar arc that crosses close to Saturn's pole from noon to midnight. A feature like this has not been seen consistently at Saturn imagery in the past but might be related to the often-seen central peak in brightness observed in ground-based observations [19,28].

The general auroral structure observed on 14 August is very similar to the H and H_2_ aurora simultaneously observed by Hubble, as described in Lamy *et al*. [49], with a strong enhancement to the dawn side main emission. The ‘bright cusp-like emission’ in the dawn–noon sector is also observed. Interestingly, although the dawn enhancement is not seen on 12 August (d), both the dawn–noon ‘cusp-like enhancement’ bifurcated spiral-shaped dusk emission do appear very similar to the Hubble observations two nights later, suggesting that these features are either recurrent or sustained over the 48 h separating these observations. On 25 July and 7 August, Hubble observed the aurora 4–8 h later than our measurements, and while the weak aurorae are similar in broad morphology, the dusk enhancement we observe on each night is not seen in these UV images [49]. This could indicate transient dusk features that are missed in the UV [11].

### (b) Velocity structure

**Figure 5.**
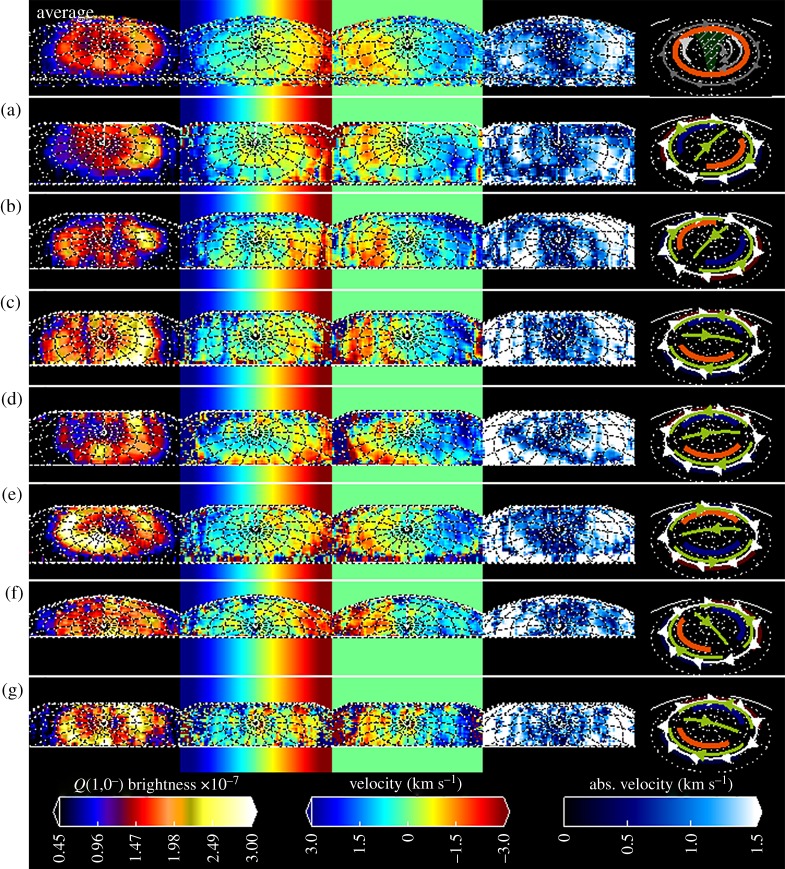
The ${H_{3}}^{+}$ brightness and ion winds. The first column shows ${H_{3}}^{+}$ emission brightness scaled between 0.45–3 × 10^−7^ Wm^−2^ µm^−1^. The second column shows the line-of-sight velocity with ranged between blue and red shifts of 3 km s^−1^. The third column shows the same velocity once the rotation rate of the planet is removed. The fourth column shows the absolute value of the winds, here scaled to 0–1.5 km s^−1^; this provides a quick view of where the planet it co-rotating (black) and significantly sub-corotating (white). The final column shows the predicted direction of ion wind flows driven by the planetary period current system based upon the average northern phase, assuming the thermosphere is driving ion winds (green), along with the location of auroral enhancement caused by this current system. Each row shows a separate night, as described in table 2, with the final row showing the average value across each night.

**Figure 6.**
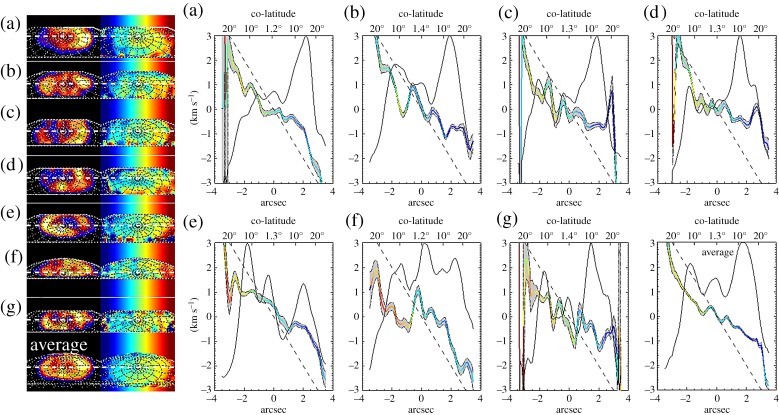
One-dimensional profiles of ${H_{3}}^{+}$ intensity and line-of-sight velocity. These are smoothed by 0.5′′ × 0.5′′. On the left are maps of intensity and velocity. The first column of maps (left) shows the ${H_{3}}^{+}$ emission scaled between 15 and 100% of the maximum intensity, using the same linear colour scale as is shown in the colour bar in figure 5 (instead scaled between 15 and 100%). The second column of maps (right) shows line-of-sight velocity, ranging between +3 and –3 km s^−1^. This velocity range has the exact scaling shown in the colour scale (figure 5). The position of the profiles is shown as a dashed white line over these. On the right, one-dimensional profiles of normalized intensity (thin line) and line-of-sight velocity (red-blue bold line) are shown. The colour of the velocity profiles is shaded by their velocities within the rotational frame of the planet, scaled between +3 and −3 km s^−1^. Velocity errors are shown in the shaded region around the velocity (grey infill). Individual profiles can be identified by the letter in the top right corner, described in table 2. (Online version in colour.)

For each night, we have calculated the line-of-sight velocities, both in the frame of the observer (effectively, the inertial frame) and the planet's rotational frame. In presenting our measurements, we use two different levels of smoothing. In figure 5, we show the unsmoothed, highest-spatial-resolution, data. In figure 6, we apply a box-car smoothing of 0.5′′ in both the *x* and *y* directions (applied to the spectra before the Gaussian fitting).

Figure 5 shows images of the ion wind velocities on each night, along with the averaged velocity across all these nights. The average is taken from these velocity maps (excluding any values where the absolute velocity exceeds 3 km s^−1^ since these values typically result from poor quality fitting). The first row in the figure is this average brightness and velocity (row ‘average’), with each individual night shown below, labelled (a–g) as described in table 2.

The first column shows the ${H_{3}}^{+}$ emission brightness, linearly scaled between 0.45 and 3.0 × 10^−7^ Wm^−2^ µm^−1^. The second column shows the line-of-sight velocity across the planet ranging between +3 (blue) and −3 (red) km s^−1^. Away from the planet, the rotation rate of Saturn is shown as a colour gradient, for context. Although the variation in Saturn's rotation rate is highly significant in understanding the longitudinal phase of the magnetic field, this variation represents a change in rotational line-of-sight velocity of less than 0.1 km s^−1^ at the auroral limb. As such here, and throughout the paper, we use the IAU rotation rate for Saturn to calculate line-of-sight co-rotation velocity. The third column shows the line-of-sight velocity in the planetary frame (with Saturn's rotation removed), again ranging between +3 and −3 km s^−1^, blue to red. The off-planet regions are green (zero velocity) in this column.

The velocity measured in the line-of-sight of the observer (column 2) clearly shows the rotation rate of the planet on each night, within the non-auroral regions, though these have significant noise associated with the weak ${H_{3}}^{+}$ emission in this region. The auroral region shows very significant ${H_{3}}^{+}$ ion wind structures every night. Some care is needed in interpreting these measurements. Purely vertical (on the figure) features—for instance, a narrow vertical blue on the dawn side of several nights (within the auroral region on (c) and, much less clearly, on the dawn flank in (e, f, g)—are instrumental errors, not related to the planet.

The line-of-sight velocities (columns 2 and 3) show significant sub-corotation across most of the polar regions for every night. These flows are more easily observed in the planetary reference frame (column 3)—red shifts on the dawn side and blue shifts on the dusk side both indicate significant sub-corotation. On average (Row ‘Average’), it is clear that strong sub-corotational flows coincide with the brightest ${H_{3}}^{+}$ emission, which itself corresponds to the main auroral oval. But strong sub-corotational flows also extend down to at least co-latitude 20°, encompassing secondary ovals that correspond to the ‘breakdown-in-corotation’ oval. At lower latitudes, the data show alternating bands of sub- and super-corotation, but these features may be due to the difficulty of fitting velocities to lower intensity ${H_{3}}^{+}$ emission, and are not discussed further.

Most nights have very significant flows on both the dawn and dusk sides of the planet. These flows, unlike past observations, are revealed as narrow arcs of flow, at a similar spatial scale as the observed auroral emission. Indeed, these flows are often observed in multiple arcs. On 25 July (a), there are two arcs in the dawn–midnight sector, one close to the main emission centred on a co-latitude of approximately 12° and the other near the pole at approximately 5°; these appear to be mirrored by offset flows on the dusk, with two clear arcs in the dusk–noon sector at approximately 15° and 20°. A similar dawn pattern is seen on 12 August, with arcs of flow again close to the pole at dawn–midnight, but these are mirrored by more general sub-corotation further out on the dusk side. Indeed, several nights show much broader regions of flow on the dusk side (b, d, e). Only a single dawn side flow is observed on 14 August (e), the night after the strongest Solar Wind activity.

In order to help clarify these flows, column 4 of figure 5 shows the *absolute* planetary frame line-of-sight velocities, scaled between 0 and 1.5 km s^−1^ (dark blue to white) to emphasize regions of lower velocity. This column is especially useful for identifying where the ionosphere appears to co-rotate with the planet. On average, there is a region extending less than 4° of co-latitude around the pole where co-rotation appears to be being enforced.

Column 5 shows the expected flows and aurora based on both the predicted current systems within the polar region. Thermospheric winds that have been predicted by examining the magnetic field oscillations within the magnetosphere are shown, for Saturn's northern phase oscillation, in green for each night [20]. The direction of these flows can be predicted from measurements of the oscillations they produce in magnetic fields within the magnetosphere, which have been used to calculate the ‘phase’ of the planetary period [55]. Each hemisphere has its own phase, both defined for each observation in table 2, rotating with the planet at the rotation rate of that hemisphere. In the northern polar region, it is expected that the thermosphere will flow over the pole towards a phase of 0°, with return flows at lower latitudes flowing back towards a phase of 180°. Also shown is the region of expected upward field-aligned currents (orange), where downwardly accelerated electrons will produce the brightest aurora), and the region of downward currents (blue). The auroral emission is not well correlated with the regions of expected enhancement caused by the planetary period current system, and is likely to be produced by more complex magnetospheric processes than current models can predict easily.

The exact positioning of flows across Saturn's polar region at all scales can be more easily resolved using one-dimensional cuts through the images (figure 6). To create these profiles, we have applied a box-car 0.5′′ × 0.5′′ smoothing to the images before fitting. Figure 6 shows one-dimensional normalized intensity and line-of-sight velocity profiles for each night at the slit position that cuts through planetary west–east/dawn–dusk at the north pole, as in previous studies (e.g. [19,28–30]). For context, we also show maps of intensity and line-of-sight velocity: the first column shows the ${H_{3}}^{+}$ *Q*(1,0^−^) line emission brightness, here scaled between the maximum intensity and 15% of that peak brightness, as in column 1 of figure 5; the second column shows the line-of-sight velocity, ranging between +3 and −3 km s^−1^, in the inertial reference frame. Overlain on the brightness and velocity images are the position of the profile for each night.

The average intensity profile (the bottom right panel of figure 6) clearly shows the two peaks of the main auroral oval around +/− 1.8′′ from the rotational pole, corresponding to approximately 12° of co-latitude. Secondary, and much less pronounced, auroral peaks are visible at lower latitudes, around +/−3.3′′ from the pole, most clearly seen on the dawn flank of the auroral-polar region. As expected from figure 5, the *average* velocity profile (figure 6, column 6) shows the broad region of sub-corotational flow extending right across the auroral polar region for at least +/− 3.5′′, equivalent to a co-latitude of 24° at Saturn, as the velocities in the profile depart from the line of co-rotation by some 2 km s^−1^ at maximum. This region of general sub-corotation clearly extends well beyond (equatorward of) the main auroral oval. There is also a region extending for about +/− 0.5′′ (+/− 5° of co-latitude) around the rotational pole where, again on average, the ionosphere is in co-rotation with the planet. This region also corresponds with a polar intensity peak; these features are discussed further in the next section.

The intensity profiles of the individual nights show considerable variability. Plots (a–d, g) show strong main oval aurorae on the dusk side, but less intensity on the dawn, while this is reversed in (e), and in (f) dawn and dusk oval intensities are roughly the same. All the plots show some evidence of a central, polar peak, with this being the strongest feature in (f). But none of the *individual* plots shows co-rotation at the pole, with—perhaps—the exception of plot (b).

On every night, multiple arcs of ion winds can be observed clearly across the pole. The strongest of these flows, seen in the images, are very clear, but there also appear to be significant smaller-scale variations across the whole polar region. The auroral brightness also shows very significant variability, and again includes smaller-scale changes within the pole. These changes may indicate continuous bands of emission and flow, or they could indicate that the regions of flow and emission change in position during the several hour exposures taken each night. Several features within this complex set of flows can be observed. Firstly, the peak emissions appear to occur equatorward of the strongest velocity flows, at least on the dawn side (a–d, g), as might be expected from current magnetospheric–ionospheric models based upon magnetospheric measurements [20]. On the dusk, the sub-corotation is typically broader, and does not correlate as well with the main emission. The flows on the dawn side are also often sharper and stronger (a, b, c and g), but the dusk side sub-corotation sometimes increases equatorward of the main emission (c, d and g), with sub-corotation velocities exceeding 3 km s^−1^ in some cases (c and d). At other times, the dusk region equatorward of the main emission has reduced sub-corotation (a, e and f), with values less than 1 km s^−1^.

### (c) Thermal structure

As already said in §2, it is possible to calculate the ${H_{3}}^{+}$ temperature, column density and total emission for each spectral position in both the *x* and *y* directions of the image using the measured brightness of the ${H_{3}}^{+}$ *Q*(1,0^−^) and *R*(2,2^−^) lines. Since the *R*(2,2^−^) line is approximately three times weaker in Saturn's auroral region than *Q*(1,0^−^), we smooth our data by 1.0′′ in both the *x* and *y* directions (applied to the data before Gaussian fitting).

**Figure 7.**
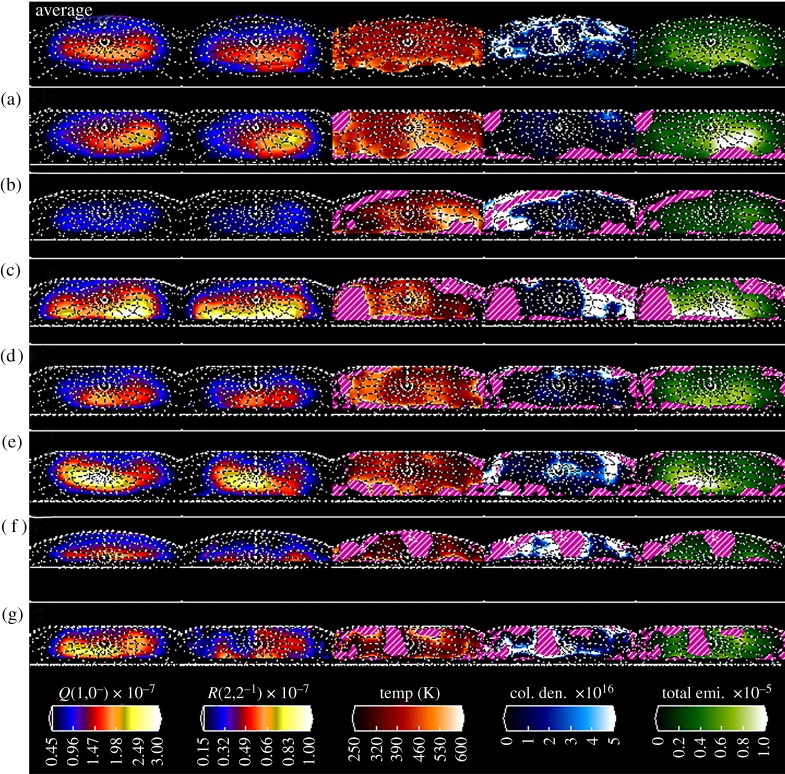
Measurements of the thermal structures observed at Saturn. These are calculated using the ratio of emission from two ${H_{3}}^{+}$ fundamental emission lines, *ν*_2_ *Q*(1,0^−^) (the left most column, shown with linear brightness scaled to a peak brightness of 3 × 10^−7^ Wm^−2^ µm^−1^) and *ν*_2_ *R*(2,2^−^) (the second column, shown with linear brightness scaled to a peak brightness of 1 × 10^−7^ Wm^−2^ µm^−1^). The third column shows the calculated ro-vibrational temperature between 300 and 650 K. Values outside this range quickly tended towards unphysical values, and so these have been shaded with pink and white stripes. The fourth column shows the column density, scaled to 1 × 10^17^ m^−3^. The final column shows the calculated total ${H_{3}}^{+}$ emission for the auroral region, scaled to 1 × 10^−5^ Wm^−2^. Each row shows a separate night, with the first row showing the average value across each night.

Maps of these parameters of the ionosphere on each night, along with the average values, are shown in figure 7. Unlike figure 5, the first column here shows the ${H_{3}}^{+}$ *Q*(1,0^−^) line emission brightness after it has been line-of-sight corrected, representing the emission radiated vertically from the planet. This is linearly scaled between 0.10 and 1.5 × 10^−7^ Wm^−2^ µm^−1^. The second column shows the ${H_{3}}^{+}$ *R*(2,2^−^) line emission brightness, again corrected for line-of-sight brightening, linearly scaled between 0.033 and 0.5 × 10^−7^ Wm^−2^ µm^−1^ (i.e. exactly one-third the brightness of column 1—the brightness of the two images match in this scaling when the temperature of the thermosphere is approx. 464 K). The third column shows the calculated ${H_{3}}^{+}$ ro-vibrational temperature scaled linearly between 250 K and 600 K. Derived temperatures outside this range tend to be unrealistic, as they result from noise rather than actual measurements of the *Q*(1,0^−^) and *R*(2,2^−^) brightness: we estimate the *Q*(1,0^−^) line within the auroral region has a S/N of approximately 20 after smoothing, falling to less than 5 within sub-auroral regions. As such, we ignore these values (and shade them with pink and white stripes in figure 7). The fourth column shows the calculated ${H_{3}}^{+}$ column density scaled between 0 and 5 × 10^16^ m^−3^. The fifth and final column show the calculated column integrated ${H_{3}}^{+}$ total emission scaled between 0 and 1 × 10^−5^ Wm^−2^, again adjusted for line-of-sight.

Looking at the temperatures, it is clear that they are most reliable inside of the 10° co-latitude circle. Outside of this circle, reliable temperatures can be derived for most of the maps (a–e) as far as co-latitude 20° (and in some cases, beyond) for the dawn and dusk segments. Maps (f) and (g) have much more patchy coverage of reliable temperatures. This then feeds into the derived column densities and integrated ${H_{3}}^{+}$ emission maps. The average maps show higher dayside temperatures and total emission in the co-latitudes (5° to 15°) that cover the main auroral ovals, although the average nightside density in this region is higher than on the dayside, a sure sign that particle precipitation is responsible for generating the ${H_{3}}^{+}$ ion. Cutting these maps across from noon to midnight, dusk temperatures, densities and overall emissions are higher on the dusk (noon through midnight) side of the planet than on the dawn (midnight-through-noon) sectors. This and the individual day maps will be discussed further below.

**Figure 8.**
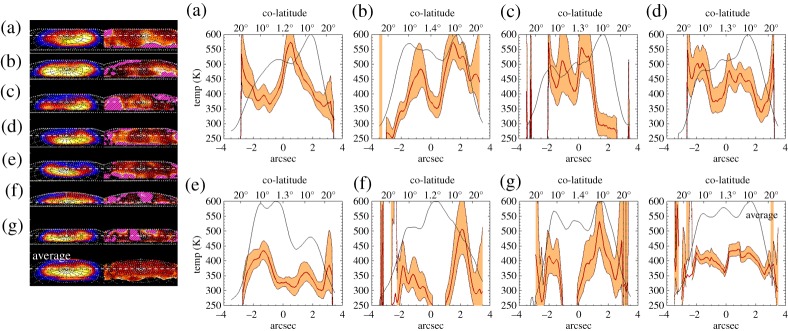
One-dimensional profiles of line-of-sight corrected ${H_{3}}^{+}$ intensity and temperature. These are smoothed by 1.0′′ × 1.0′′. On the left are maps of intensity and temperature. The first column of maps shows the line-of-sight corrected ${H_{3}}^{+}$ emission scaled between 15 and 100% of the maximum intensity, again using the same linear colour scale as is shown in the intensity colour bar in figure 7 (instead scaled between 15 and 100%). The second column of maps shows temperature ranging between 250 and 600 K, using the exact same temperature scale as is shown in figure 7. The position of the profiles is shown as a dashed white line over these. On the right, 1-d profiles of normalized line-of-sight corrected intensity (thin line) and temperature (bold red line) are shown. Temperature errors are shown in the shaded region around the temperature (orange infill). Individual profiles can be identified by the letter in the top right corner, described in table 2. (Online version in colour.)

The extent of small spatial scale thermal variability is revealed further in one-dimensional cuts through these images. In figure 8, we show one-dimensional profiles of emission and temperature cutting through the auroral region, with temperatures bound by the calculated errors in temperature. The first column shows the line-of-sight corrected ${H_{3}}^{+}$ *Q*(1,0^−^) line emission brightness, scaled between the maximum intensity and 15% of that peak brightness. The second column shows the calculated temperature, ranging between 250 K and 600 K, in exactly the same way as column two of figure 7. We take cuts through these spectral images at the same location as those shown in the velocity profiles in figure 6. Overlain on the brightness and velocity images are the position of the cut for each night shown in the rest of the figure; there is significant small-scale structure that is not easily seen in the temperature maps.

For the average plot, temperatures range from approximately 350 K to 450 K across the main auroral oval region. The polar intensity (central) peak corresponds to temperatures at the lower end of this range, while the main auroral oval has temperatures closer to 430 K. Noticeably, the central peak in this plot corresponds to a noon–midnight ‘bar’ of higher density in the average density shown in figure 7.

Within individual nights, large temperature differences occur over small distances, with temperatures changing by as much as 300 K in some nights (c, f, g). However, even in regions that have more stable temperatures, there appears to be significant structure, with temperature oscillations across a wide range of values, but clearly defined at ranges as small as 30 K (a, d and f all have regions with these small variations), though these variations are smaller than the calculated errors. Most notable, again, is the structure of the average temperatures, where the temperatures can be seen to have a consistent temperature of approximately 420 K across the auroral region, dropping off at the edge of the aurora to temperatures of approximately 350 K. Also of interest is that there is a clear region at the pole of the planet where the temperature is approximately 50 K lower.

It is worth remembering, however, that we use only two emission lines in calculating the ${H_{3}}^{+}$ temperature, with no redundancy. This makes it difficult to fully understand systematic errors on temperature retrieval, such as non-LTE conditions, or the effects of atmospheric temperature gradients. This may help explain some of the extreme temperature changes observed here, and so future observation using multiple emission lines are needed to fully understand the causes of the large-scale temperature variability observed here.

## 4. Discussion

### (a) Average behaviour of Saturn's auroral-polar regions

From §3, we can make a number of generalizations about the average behaviour of Saturn's auroral-polar ionosphere, as measured during the month from 25 July to 25 August 2017.

— The brightest ${H_{3}}^{+}$ emission is located around the poles between co-latitudes 10° and 15°. The emission structure forms a clear auroral oval, positioned at a co-latitude of approximately 12°. Inside this, a central arc of emission extends from noon to midnight on the dawn side of the planet.
— Temperatures range from approximately 350 K to 450 K across the main auroral oval region. The main oval temperature is around 430 K. The average maps show higher dayside temperatures and total emission in the co-latitudes (5° to 15°) that cover the main auroral ovals, although the average nightside density in this region is higher than on the dayside.
— Looking from noon to midnight, dusk temperatures, densities and overall emissions are higher on the dusk (noon-through-midnight) side of the planet than on the dawn (midnight-through-noon) sectors.

Past measurements of the average auroral emission using the Cassini VIMS instrument show very similar intensity structure [64]. Those observations were made using a more extended dataset (greater than 60 images in the north) and at a higher spatial resolution, but with a much lower spectral resolution, so that background reflected sunlight is potentially more problematic. In those measurements, the main oval is located at approximately 15° on the dayside, close to our measured location, but perhaps indicating our observations show a slightly more contracted auroral oval. Focusing on the averaged emission and velocity maps (shown in the bottom row), we find several very interesting structures. These maps provide a much better view of the local time-average flows, consisting of a wide range of planetary period phases, including several that are in anti-phase with one another. As such, they provide a background local-time fixed ionosphere, over which the more complex small-scale arcs of emission and ion flows observed on each night are overlaid.

— On average, strong sub-corotational flows coincide with the brightest ${H_{3}}^{+}$ emission, which itself corresponds to the main open-closed field line boundary auroral oval, with some localized flows in dawn and dusk between 5° and 10°.
— But strong sub-corotational flows also extend down to at least 20°, encompassing secondary ovals that correspond to the ‘breakdown-in-corotation’ oval.

The similarity between the mean emission structure in our observations and those previously observed suggests that the auroral conditions observed during the Grand Finale are representative of the conditions more generally. In this context, we can assume that the ion winds we measure are also representative of typical local time fixed ion flows. The line-of-sight velocity shows clear sub-corotation directly associated with the auroral-polar region, with co-rotation at latitudes lower than this.

— There is a region extending less than 4° of co-latitude around the pole where co-rotation appears to be being enforced. The polar intensity (central) peak corresponds to temperatures around 350 K. The central peak in this plot corresponds to a noon–midnight ‘bar’ of higher density.

The central region of co-rotating plasma extends across the pole from noon to midnight, appearing to flare out wider away from the Sun. It is not clear how this morphology relates to the current theory of a central core of older field lines rotationally twisted, shielding the centre from reconnection. But closed field lines that are extending far into the tail near midnight are unlikely to strongly affect the ionosphere. Interestingly, the location of the noon–midnight arc of emission appears to be closely co-located with the dawn edge of this co-rotational region.

Past VIMS observations also show a clear region of enhanced emission extending across the northern pole on the dawn side [64]. Although this is not as clearly an arc of emission, it is also brightest in the noon–dawn sector—the observations of the southern aurora also show this emission, and there it can be seen to extend to the midnight side of the main oval. Interestingly, these arcs are much more dawnward in the VIMS observations. Such polar arcs are rarely seen in individual VIMS images (e.g. [18]), but are much more common in ground-based observations, seen as a central peak over the pole. Here, we show that this central feature is an extended arc of emission that crosses the pole from approximately noon to midnight and that, while only seen in VIMS data once multiple nights are co-added, is actually quite common.

These arcs may be similar to the polar auroral filaments sometimes observed at Jupiter, which are also fixed in local time and extend from noon outwards, and have been associated with magnetic field lines that map far into the outer magnetosphere [65]. These are typically fixed in local time near noon, but can move significantly nearer midnight, a feature perhaps supported in our average emission maps, since the arc broadens towards midnight. These could also be associated with the theta aurora seen at Earth, though as Nichols *et al*. [65] point out, an Earth-like mechanism for producing transpolar arcs would be difficult in the single-celled convection pattern across the pole expected in the rotationally dominated ionospheres of both Jupiter and Saturn.

In figure 5 column 5, we showed a schematic summary of the observed auroral and ion wind flow structures. The average auroral flows observed here clearly need to be considered carefully in future models, and reveal significant problems with the past axisymmetric approach to modelling these flows.

### (b) Night-by-night observations

Over the first two weeks of observation, the general morphology of the ${H_{3}}^{+}$ aurora appears to be somewhat stable, with a significant number of nights that are brighter on the dusk (a–d, f). Although extended periods of dusk brightening are unusual, with dawn brightening usually dominating, they are not unprecedented [25]. What is compelling here is that the auroral emission and ion wind structures observed are very familiar, both in previously published IRTF observations [19,28,29,32,63] and in the broader database of observations taken between 2003 and 2016. Several nights still show a clear three-tier velocity structure (b and g most clearly), there is clear sub-corotation across the entire pole, and the emission structure shows two or three peaks. The small-scale flows seen in our Keck observations are likely to have been occurring in past observations, obscured only by the smoothing and increased noise within that data.

The most significant change over the entire month is the very significant auroral brightening on 14 August, which produced a strong dawn enhancement (e). These morphologies are well within the range of different structures previously observed for Saturn's aurora, and although the strong dawn enhancement on 14 August does suggest significant changes in the magnetospheric conditions, it does not match with the strongest auroral responses previously observed, where the entire dawn region of the aurora is infilled [24]—simultaneous Hubble observations provide a similar conclusion [49].

While some ‘sub-corotational’ arcs that we have described earlier lie close enough to the centre of the planet that they could indeed be transpolar, there is no clear evidence of this—indeed these arcs do seem to follow lines of latitude. There are no clear flows at the meridian. This is in clear contrast with recent analysis by Chowdhury *et al*. [30], which shows a strong noon–midnight flow over the rotational pole. The size of the region of co-rotation in the pole is often significantly larger than can be explained by line-of-sight effects. This results in a region of co-rotation that is sometimes only a few degrees wide (a, b, f), but can be much more significant (c, e). This is the manifestation of the ‘three-tier’ velocity structure seen in past observations [29]. Notably, this region of co-rotation is well confined in the dawn–dusk region, but can extend much further in the noon–midnight direction. Interestingly, this includes 14 August (e), where the enhanced Solar Wind strength might have been expected to have the strongest effect. However, the emission structure for this night does not have the very clear dawn-infilled structure seen in past of observations of these most extreme of Solar Wind interactions [24], and, so, although some re-connection is likely, the expected complete closure of old, co-rotating, open field lines is unlikely to have occurred here [32], a conclusion that is borne out in the velocity structure, which shows clear co-rotation in the polar region. There are only two nights where clear central co-rotation regions are not observed. On 12 August (d), the multiple arcs of sub-corotation extend all the way to the pole, leaving no clear region which co-rotates. On the 25th August (g), the velocity structure is very fragmented, possibly due to the short integration time, but appears to show similar cross polar arcs of sub-corotation.

Column 5 of figure 5 shows the expected flows and aurora based on both the two predicted current systems within the polar region. The auroral emission is not well correlated with the regions of expected enhancement caused by the planetary period current system (shown as an arc of orange in column 5), and are likely to be produced by more complex magnetospheric processes than current models can predict easily. To understand the expected ionospheric flows from these models, the calculated flows associated with the planetary period current system (shown in green), would be observed combined with the local-time fixed sub-corotation associated with the outer magnetosphere (shown as a white ring of flow against rotation). Where these flows are in the same direction, we would expect stronger sub-corotational ion winds, and where they are oppositely directed, we would observe co-rotation. Importantly, because the planetary period wind system is a twin-cell structure, this should split the auroral region into two halves, one with strong sub-corotation and the other with more co-rotational flows. We can compare these regions of combined and counter flows against the observed sub-corotations highlighted in columns three and four. When the magnetic phase is close to midnight, as on 25 July (a), much stronger sub-corotation on the dusk than the dawn is expected. Although there is a slight enhancement between noon and dusk, it is matched by flows that are almost as strong between midnight and dawn. The flows on 1 August (b) should be oppositely directed, but there is no notable difference between these nights in the flow strength on each side of the auroral region. The orientation on 20 August (f) has the only clear example that seems to show a bias between the dawn and dusk flows, and here the strong flows on the dawn side do agree with the proposed ionospheric flows. Each of the other nights (c, d, e and g) sees the strongest modelled flow differences occurring perpendicular to the line-of-sight, so should be less effective in showing these flows. However, in each case, a significant difference in the noon–midnight extent of sub-corotation should be seen, yet each of these nights shows very little change in flow over this region. We also considered whether the planetary period currents that result from the southern auroral region might be interfering with this system. The southern current system would best interfere with the northern system if the phases of the two systems, as described in table 2 were similar. Our observing period was long enough for the phases of the two hemispheres to go from being approximately in phase, to drifting into anti-phase, then back in to phase again. As such, for several of our observations (c, d, e and f) the southern planetary period current system should have acted to enhance the northern current. All this suggests that we cannot see the effect of the planetary period currents within the atmosphere, let alone show whether these flows are in the direction predicted by models that evoke an atmospheric origin for these currents. This perhaps suggests this period is dominated by other auroral processes, for instance, the significant Solar Wind activity observed in this period. However, there are clearly non-axisymmetric flows in our data, which could well be indicating an underlying influence from the planetary period current. The only way to properly confirm whether this is the case would be to recombine the flow measurements into planetary period phase rather than local time, but this is beyond the scope of this paper.

Although two nights where the dusk side is strongly sub-rotating (d and g) show a slight enhancement on the equatorward edge of the dusk-side main emission, there is no evidence of the significant poleward extending bifurcations in the dusk side aurora sometimes observed [66]. The extended region of sub-corotation observed on the dusk side of the aurora might indicate that the dusk side polar region is dominated by Vasyliunas cycle driven periodic breaking off of plasma in the dusk magnetosphere. The changes in extent of sub-corotation on different nights may be the result of switching between gentle and more extreme sub-corotation in the ionosphere. Although changes in the magnetotail have been shown to be quasi-periodic with the planet's rotation rate, reconnection does not occur on every rotation [67], and this periodic behaviour can also be affected by phase-shifts and non-rotational frequencies [68]. It may be that the currents that drive these bifurcations occur readily in the dusk side, but that the dusk side of Saturn's aurora only results in bright arcs of emission under the right conditions, where currents are strong enough to drive accelerated elections into the atmosphere. In contrast with this, the dawn side may be revealing the return flows of either the Vasyliunas or Dungey cycles, squeezed as these return onto the dayside of the magnetosphere, resulting in more significant flows. This broadly agrees with past modelling of the single-cell local-time-fixed flow system associated with a rotationally dominated Dungey cycle at Saturn [69].

It may be noteworthy that the velocities measured often appear to occur in quartile sectors. Most notable of these are the flows on 25 July (a), where twin arcs of flow are seen in the dawn–midnight and dusk–noon sectors, but very little flow is observed in the dawn–noon and dusk–midnight sectors. Current magnetospheric and thermospheric modelling of Saturn fails to predict such a quartile velocity structure. Modelling at Earth has shown that ion winds driven anti-sunward by the Dungey cycle during active aurora can drag the thermosphere into complex morphology, producing three cyclones and three anti-cyclones, driven by three hot spots and three cool spots [70], perhaps hinting that thermosphere–ionosphere interactions might be the source of this complex structure.

Interestingly, there also appears to be a significant noon to midnight gradient in temperature, with noon being approximately 80 K hotter than midnight. This is the reverse of the temperature gradient observed by Keck in 2013 [45]. Given the difference with what was observed in the past, this suggests that rather than increased conductivity or solar heating, this gradient results from an increased level of auroral energy on the noon side of the aurora during this period.

The most notable aspect of these observations is the significant variability observed in the temperatures within the ionosphere. Although some past observations showed that the temperatures could change by as much as 150 K in only approximately 20° of rotation [45], the degree of variability seen here is still very significant. Temperatures across the auroral region appear to change by more than 200 K over relatively small spatial scales. More than this, the regions that show enhancement are in no way consistent over the entire month. Within some observations, the dusk is hotter (a and b), some appear hotter in the auroral region (d and e), while on 7 August (c) the ionosphere is hottest over the pole and the final nights are generally much cooler (f and g). Notably, the consistent temperature structure appears in pairs, suggesting that the broad-scale thermal changes may be occurring on a timescale of at least approximately 50 h (the time gap between d and e), and possibly as long as 14 Earth days (the length between a and b).

The thermal structure observed here indicates one of two origins. The temperatures measured may reveal underlying thermal structure in the thermosphere, resulting from changes in auroral heating or other thermospheric heat sources. Alternatively, the changes in temperature may indicate a change in the altitude of peak ${H_{3}}^{+}$ ionization, coupled with the increase in thermospheric temperature with altitude; higher layers of ionization would be coupled with a hotter thermosphere, while deeper layers of ionization would be heated to cooler temperatures.

Significant thermal differences are expected if the thermosphere is driving the ionosphere [39], but when comparing with the northern planetary phase shown in figure 5, there do not appear to be any significant regions of heating that are consistent with any phases of the aurora. Column 4 shows the measured values for the ${H_{3}}^{+}$ column density, which appears highly variable. Although these values are the correct magnitude of column density values, their variance, at least on individual nights, are too large to provide detailed understanding of the sources of precipitation. However, on individual nights, there are no clear alignments between column density and temperature, suggesting that particle precipitation is unlikely to be a primary source of these small-scale temperature changes. Similarly, comparisons between the temperatures and the regions where the sub-corotations are strongest show no significant correlations, with some of the nights that have symmetric sub-corotational flows having highly asymmetric temperatures (a and b), while those with asymmetric flows typically having balanced temperatures (d and f). This suggests that the temperatures are not dominated by Joule heating. Comparison between the temperatures and total emission appears to show the ${H_{3}}^{+}$ emission is strongly correlated to temperature, but that this cooling is ineffective at changing the thermospheric temperatures; this is in direct contrast with Jupiter, whose ${H_{3}}^{+}$ is a strong thermostat in the upper atmosphere, modulating temperatures in the ${H_{3}}^{+}$ region of the atmosphere [71]. The auroral region does appear to cool significantly after the initial arrival of the compressed Solar Wind; increased auroral heating might be expected to drive temperatures up, but the drop in temperatures after 14 August (f and g) suggest ${H_{3}}^{+}$ may have acted as a more efficient thermostat during this period of extended Solar Wind compression.

Alternatively, the localized temperature variability may be controlled by variations in particle precipitation flux, resulting in the formation of a peak ${H_{3}}^{+}$ emission at different altitudes, and thus at different thermospheric temperatures. Indeed, the strong correlation between the average ${H_{3}}^{+}$ emission structure and temperature and lack of correlation with density seems to imply that the observed temperature structure is mostly an altitude effect. However, while current models do not provide an accurate measure of the altitudinal thermal structure within the thermosphere, temperature changes of approximately 300 K are likely to need very significant changes in peak ionization, perhaps several hundred kilometres. Past observations of the UV have shown changes on this scale [72], but ${H_{3}}^{+}$ is likely to be more constrained in altitude, as deeper penetrating electrons will fall below the homopause, leading to the destruction of any ${H_{3}}^{+}$H_3_^+^ generated. In addition, recent analysis of the vertical thermal profile at Saturn [73], has shown the temperature does not change significantly with altitude within the thermosphere. In addition, the correlation observed between temperature maps on successive individual nights, separated by several days, is also very difficult to explain by altitude effects, given the dramatically different auroral emission (and so, most probably, auroral electron flux) on these nights.

All this suggests that localized temperature variability is most defined by processes that have not yet been considered, or, more likely, the interplay of multiple processes. When analysing the mean values for these physical parameters over the month of observations, an interesting pattern is revealed. Despite significant variations on a nightly time scale, the temperatures smooth out significantly over the month. In these average temperatures, the range of values is too small to discuss without rescaling, as we do in the following two figures. However, both column density and total emission also appear to be closely aligned with the ${H_{3}}^{+}$ emission.

## 5. Conclusion

Our observations of Saturn's auroral region during the final month of the Cassini mission have provided the first ever maps of ionospheric flows and energy sources. These observations are also unique in the solar system, providing detailed views of the ionosphere after the surrounding space environment has been measured and modelled in detail. As a result, our models of Saturn's magnetic fields and the currents systems within its magnetosphere have been mapped into Saturn's ionosphere without sufficient details within the ionosphere and thermosphere to limit these models at the planetary end of the magnetic field lines. As a result of this unique position, our observations have revealed unexpected complexity within the ionosphere that cannot be explained by any current models of magnetosphere–ionosphere interactions, and suggest that relationships between Saturn's auroral field-aligned currents, measured above the planet by Cassini, and the Pedersen and Hall currents that then close through the atmosphere, are highly complex.

Similarly, current models of Gas Giant ionosphere–thermosphere interactions are highly limited. Some model the magnetospheric interaction in detail, but simply use the thermosphere as a driver of currents (e.g. [37,38]). Past models of Jupiter and Saturn provide a full three-dimensional treatment of the ionosphere–thermosphere coupling, but are both too coarsely gridded in the auroral region and do not use measured ion winds across the pole (e.g. [74–76]). More recent modelling of Saturn's ionosphere–thermosphere coupling has revealed important information about the meridional flow of energy, but these models are typically axisymmetric, and cannot provide information at the level of detail our observations now reveal (e.g. [77,78]). Unlike Gas Giants, where thermospheric coupling is evoked to explain the major current systems that drive the aurora, the coupling between Earth's ionosphere and thermosphere is largely driven by magnetospheric forcing. However, recent models have shown that thermospheric interactions between pressure gradiences, ion and viscous drag forces and Coriolis forces are divergent, resulting in sustained thermospheric flows, leading to adiabatic heating or cooling and the creation of neutral temperature structures [79]. If Saturn's auroral processes drive thermospheric flows and localized heating, this will result in changes to the neutral driving of magnetospheric currents, whether that system is fixed in local time or rotating at the planetary period. The resulting feedback between the thermospheric flows and magnetospheric currents may help explain the high level of complexity seen in both the ion wind and temperatures in Saturn's auroral region.

Finally, these observations are likely to have more to tell with future analysis. In combining all seven nights into a local-time average, we have revealed clear ionospheric structures that are obscured in the complexity of individual nights. Our next investigation will combine individual spectra into their planetary period phase, allowing us to average the data with the rotation of the magnetosphere. This should reveal underlying thermal and ion wind structures, providing the first clear picture of how the ionosphere moves in relation to planetary period currents, what drives these currents and whether the thermosphere is the ultimate source of this current system.

## Data accessibility

The Keck data are available on the Keck Observatory Archive: https://www2.keck.hawaii.edu/koa/public/koa.php. The Cassini/MIMI data are available through NASA's Planetary Data System https://pds-ppi.igpp.ucla.edu/mission/Cassini-Huygens/CO/MIMI.
